# Biodefense Oriented Genomic-Based Pathogen Classification Systems: Challenges and Opportunities

**DOI:** 10.4172/2157-2526.1000113

**Published:** 2012-03-16

**Authors:** Willy A Valdivia-Granda

**Affiliations:** Orion Integrated Biosciences Inc., New York, USA

## Abstract

Countermeasures that will effectively prevent or diminish the impact of a biological attack will depend on the rapid and accurate generation and analysis of genomic information. Because of their increasing level of sensitivity, rapidly decreasing cost, and their ability to effectively interrogate the genomes of previously unknown organisms, Next Generation Sequencing (NGS) technologies are revolutionizing the biological sciences. However, the exponential accumulation microbial data is equally outpacing the computational performance of existing analytical tools in their ability to translate DNA information into reliable detection, prophylactic and therapeutic countermeasures. It is now evident that the bottleneck for next-generation sequence data analysis will not be solved simply by scaling up our computational resources, but rather accomplished by implementing novel biodefense-oriented algorithms that overcome exiting vulnerabilities of speed, sensitivity and accuracy. Considering these circumstances, this document highlights the challenges and opportunities that biodefense stakeholders must consider in order to exploit more efficiently genomic information and translate this data into integrated countermeasures. The document overviews different genome analysis methods and explains concepts of DNA fingerprints, motif fingerprints, genomic barcodes and genomic signatures. A series of recommendations to promote genomics and bioinformatics as an effective form of deterrence and a valuable scientific platform for rapid technological insertion of detection, prophylactic, therapeutic countermeasures are discussed.

## Introduction

Naturally occurring pathogens continue to persist, emerge and reemerge globally as rampaging menaces responsible for more than fifteen million deaths annually. Their impact on global health and economy is widely recognized. For this reason, the risk of renewed bioweapon development and the intentional use of microbes to harm humans, animals and plants is one of the most serious security issues that the U.S. confronts. This threat is exacerbated by scientific and technological advances that facilitates state and non-state actors to design and synthesize bioweapons with enhanced virulence and antimicrobial resistance and capable of avoiding exiting detection and characterization capabilities [1].

In 2009, the U.S. government released a National Strategy for Countering Biothreats (NSCB) highlighting a complementary strategy to Homeland Security Presidential Directives (HSPD) 8, 10 and 21 [2]. According to these documents, obtaining timely and accurate insights on current and emerging biothreats requires: 1) Building global capacity for disease detection surveillance, 2) Improving intelligence and the collection of data regarding the potential possession, transfer and use of biological weapons, 3) Developing and expanding the ability to produce forensic and attribution methods to prosecute the perpetrator(s) of a biological attack, and 4) Understanding the direction of new scientific progress that can lead to a new generation of biological threats. While there has been progress in science and technology investments for biodefense, several critics have questioned the return for investment in these programs. Recently, a commission on weapons of mass destruction highlighted significant vulnerabilities in our ability to discriminate pathogens and the need for more rapid development of therapeutic and prophylactic countermeasures.

The advent of next generation DNA sequencing (NGS) represents a technological capability gain to address the requirements outlined by HSPD and NSCB. Because the coverage, speed and decreasing cost, next generation genome sequencing is revolutionizing clinical, environmental and experimental microbial surveys and it is allowing the discovery of previously unknown pathogens’ genomes [3–5]. At the genomic level, next generation DNA sequencing is providing better understanding of the events of quasi-species distribution, DNA rearrangements and assortment, including lateral and horizontal DNA transfer. NGS is also facilitating the understating of the molecular evolutionary mechanisms shaping virulence, pathogenesis, antimicrobial resistance and the determinants of pathogen spread. At the metagenomic scale, data from NGS has far reaching implications [6].

As highlighted by the US Institute of Medicine, the next major infectious disease outbreak may, like HIV and SARS, be a previously unrecognized pathogen emerging in highly populated areas. It is widely expected that metagenomics will be used to identify such previously unrecognized pathogens crossing the species boundaries [7,8]. This technology also might avert and reduce the risk from the deliberate or accidental release of biological agents, including multidrug resistant pathogens. Furthermore, the development of international metagenomics projects not only will strengthen biodefense collaborations abroad but could also represent a new type of signal for surveillance and strengthen epidemiological analysis.

Despite the obvious potential of this new technology, both government regulatory agencies and researchers must confront the challenge of how to determine which dual-use technologies, such as the release of the genomic information of pathogens, have serious implications for biosecurity. Consequently, there is a need for constantly assess the risk of publishing the genomes of previously unknown pathogenic microbes that are currently poorly understood and are as yet not included within the biothreat select agent lists. Considering the dynamics with which new pathogens are being discovered, NGS is clearly pointing to the imminent need for our rethinking of classification schemes that facilitate the inclusion or exclusion of newly discovered microbes within the select agent list. However, genome-based select agent inclusion and characterization is still currently unfeasible [9]. This view arises from the fact that genomic information does not necessarily represent the tempero-spatial diversity existing in nature. This is complicated by the limitations of existing bioinformatics analysis tools in their ability to provide reliable estimates of risk. This situation is significantly constraining the rapid technological insertion of point-of-care and stand-off detection devices and the cross validation and integration of genomic information into biosurveillance programs.

With all this in mind, this article explores the challenges, limitations and opportunities of genomic analysis algorithms and their impact in countermeasure development, and, in particular, pathogen discrimination. This document also proposes new directions for the generation and use of genomic signatures and motif fingerprints as rapid and reliable pathogen discriminatory regions that can be incorporated in different countermeasures.

## Limitations of Biodefense-Oriented Genomic and Metagenomic Analysis

A major vulnerability in biodefense has been the very limited number of computational tools available for comparative genome analysis that translate this information into robust countermeasures. While various statistical, computational, phylogenetic and data mining techniques have been proposed to classify species and generate targets, the needs for biodefense require advanced discrimination systems with a resolution far beyond our current state of technological development [10]. This limitation continues to persist, despite the fact that for years there has been a need for accurate pathogen discrimination tools that use genomic and metagenomic information for biodefense purposes. Initial government investments on biodefense yielded sequence analysis algorithms that reused implementations developed in the late 1990’s or early 2000’s. Currently, 95% of genome analysis techniques used for microbial classification are based on five general classes of computational algorithms: 1) pairwise heuristic local or global homology sequence comparisons such as BLAST, FASTA and SSEARCH 2) profile-based searches such as HMM, MEME and pattern-hit-initiated BLAST (PHI-BLAST) or SCANPROSITE. 3) Phylogenetic analysis using neighbour-joining, maximum parsimony or likelihood distance functions. 4) Nucleotide pattern analysis based on use of dior tri-nucleotide frequencies and suffix tree scanning. Despite their wide use, these bioinformatics methods are woefully unsuitable for biodefense applications such as the development of detection reagents, or prophylactic and therapeutic countermeasures.

The high false positive or false negative rates of existing genomic analysis methods is due to their susceptibility to the errors and overrepresentation of sequence information for a given taxa in the query database (e.g., Influenza vs. Arenavirus). In fact, approximately 45% of the all the sequence information available for pathogenic microbes is redundant and more than 60% of it cannot be traced reliably to a specific host. For some pathogenic viruses, the error rate for serotype assignment in public databases can reach up to 12%. Given these complexities, the discrimination of closely related virulent and non-virulent strains, genetically modified or combinatorial synthetic pathogens from naturally occurring microbes is difficult. Since these methods lack the ability to detect erroneous records in reference databases, they cannot estimate with confidence the true diversity composition of a metagenomic sample. Furthermore, these methods are unable to determine if the presence of specific ‘metagenomic sequence read’ points to the causative agent of a biological attack.

### Pairwise heuristic homology sequence comparisons

**Figure 1:**
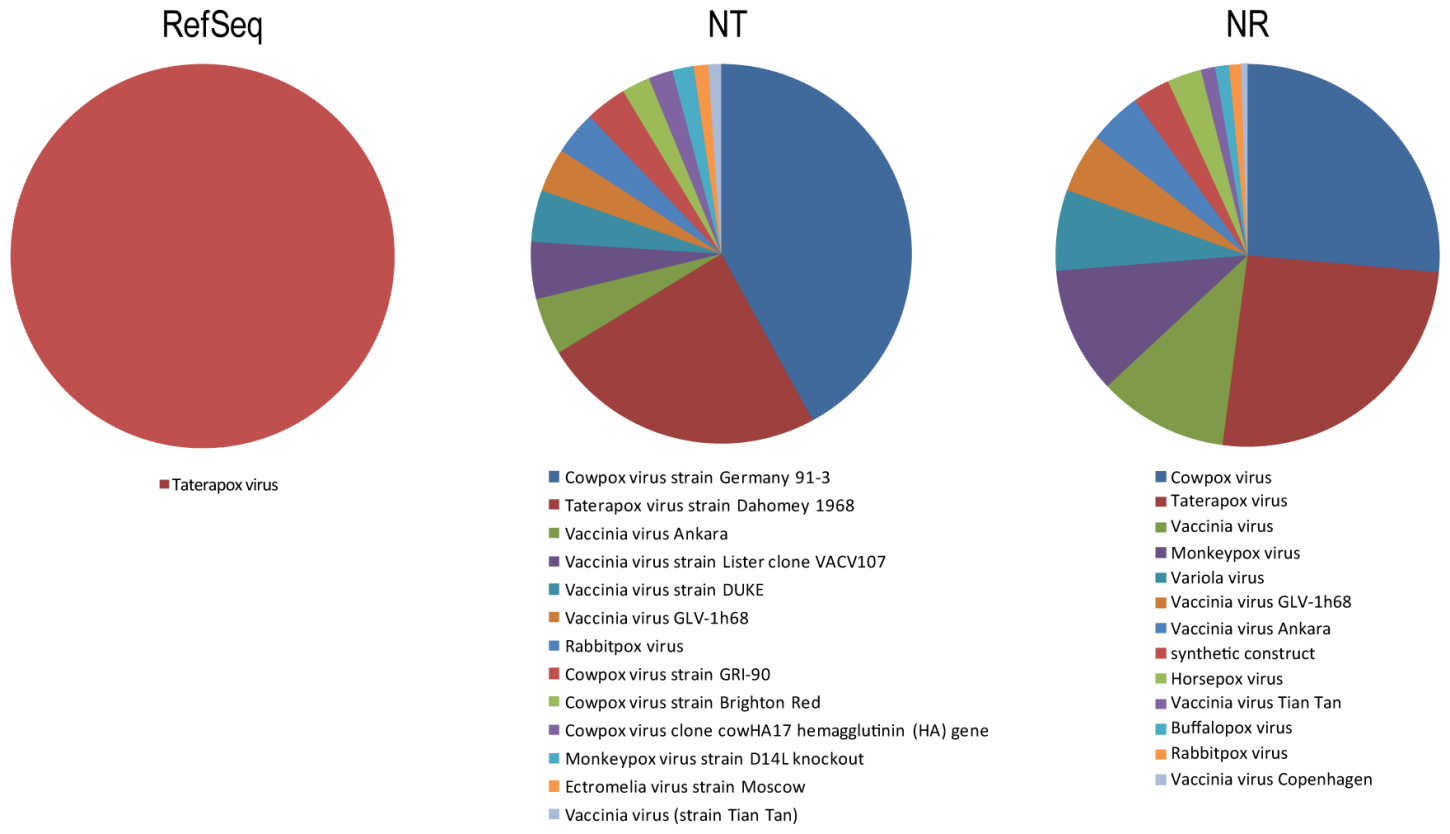
This figure depicts the impact on the assignment of species name using BLAST algorithm and different databases. While RefSeq annotated 100% of the data as Taterapox virus, the analysis of the same data with different databases pointed that the error rate and discrepancy across databases could read 80% of the initial dataset.

These classes of widely used methods apparently offer the best time/accuracy performance for unknown sequence identification and species diversity measurement [11–15]. However, FASTA, BLAST and SSEARCH use an empirical correction derived from a theoretical probability dependent on the size of the search database, this userdefined homology cut-off parameter will provide different results depending on the query sequence length and the size of the reference database. Because the best set of parameters for one part of the alignment may not be the best for another part, these software tools are unable to truly detect all homologs, orthologs, or paralogs of an unknown sequence. As a result, short DNA segments or amino acid domain regions will generate significant numbers of “false positives” that will affect the species diversity estimation. Extensive benchmarking shows that these tools will require significant computational resources when processing a single metagenomic sample and the response time using ~70 CPUs will take from 8 to 12 days. More importantly, depending of the query database (nucleotide vs. protein or nr vs. RefSeq), their false negative rate is around 15% and false positive error rate can reach 35% to 80% (Figure 1). Since genomic information is accumulating at an exponential rate, the analysis results are very unreliable and contradictory results will be obtained as new sequences are added into the system.

To address the issue of speed, in the past firmware implementations of pairwise sequence homology algorithms have been reported as reconfigurable ASIC logic (TimeLogic BLAST), CUDA compatible GPU cards (GPU-BLAST) and reconfigurable computing Field Programmable Gate Arrays (FPGA-BLAST). While achieving an average of 20-fold speed accelerations over single CPU units, these methods are not suitable for biothreat discrimination. It is important to highlight that these methods are currently widely used in the biodefense community to design PCR primers and probes, select antibodies and prioritize prophylactic and therapeutic targets; however, it now evident that there is a need for an initiative to validate and verify the potentially faulty information generated with these tools.

### Profile-based methods

These probabilistic models are implemented in hidden Markov models (HMM3) and Shannon entropy mathematics (MEME) [16–18]. These statistical and computational implementations have a strong theoretical basis in probability and are supported by algorithms for training, database searching and sequence alignment. For example, the PHMMER program is analogous to BLASTP. It takes a single protein sequence as an input query and searches it against a target sequence database by converting the query sequence into a profile HMM. One drawback to modeling proteins using HMMs is that the programs contain many free parameters and therefore require a large amount of training data and significant computational resources [18]. PSI-BLAST employs intermediate sequences in detecting remote relationships between proteins and includes in some cases multiple search iterations from an initial set of homologues for a protein in a ‘first generation’ search by querying a database [19]. However, in some cases profile-based methods could suffer from errors if the alignment incorrectly includes unrelated sequences or regions with highly biased amino acid compositions. In this case, the profile scores will produce spurious and misleading results. This is a major source of deceptive results that limits profile-based diversity measurements in known and unknown pathogen characterization. Furthermore, in many cases, the user must specify whether or not to continue the search iteration process, which will be different for each profile.

An amino acid or nucleotide profile implicitly assumes that all the aligned sequences belong to the same family or share similarity. Motifs may be represented as regular expressions or, more generally, as profiles or position-specific scoring matrices, in which each column in the matrix represents a distribution across the amino acids at that position in the motif [18,20]. To build these profiles, detailed knowledge on the input samples is required, and this availability is not necessarily the case with pathogens of biodefense relevance. In addition, many motifs discovered by this approach are poorly conserved and might not be associated with any taxonomic relevant parameter required to include or exclude virulent from avirulent strains. Considering the rate of lateral and horizontal genome exchange of some pathogens, the application of these methods to estimate diversity becomes difficult. Furthermore, the generation of profiles from some unrepresented protein families might not coincide with the mutational landscape observed in unknown pathogens present in a metagenomic sample. Finally, finding a multiple sequence alignment for long regions and building profiles from this is so computationally intensive that it is often impractical to do in real scenarios.

### Nucleotide frequency based analysis

These implementations are based on the theory developed in the 50’s by Chargaff’s four rules of DNA base composition [21]. This approach assumes that each organism has a unique evidence signature defined by the species-specific frequencies of short oligonucleotides. This search process is established first by shifting the search frame one base or amino acid at a time and storing this information in a look-up table and performing associations of an unknown sequence to a specific frequency of nucleotides or amino acids (k-value) [22,23]. However, in some cases this method can yield erroneous results when recognizing codon-optimized genome segments that deviate slightly from the reference frequencies. The bias of these methods towards the compositional heterogeneity produced by changes in the number and length of the input sequences makes this approach unreliable for recognizing pathogens with particular genome information. Also, the higher the k-value, the more information is captured; however, as the k-value increases, this is counterbalanced by the background random noise. As a result, these methods perform well with k>3. However, at this scale, these methods cannot identify previously unknown species or discriminate closely related strains. While some methods have used suffix trees [24] nucleotide frequencies codon optimization nucleotide frequency composition cannot be established as a reliable pathogen discrimination, as bootstrapping is not possible and it is necessary to correct for different genome sizes [25,26].

### Phylogenetic-based pathogen discrimination

Approaches are very subjective and impractical for pathogen characterization, attribution and forensics because the analysis requires iterative distance calculations that are time-consuming and that generally produce contradictory results [27]. Phylogenetic approaches infer erroneous classifications due to nucleotide convergences between isolates belonging to different lineages that join accidentally by a particular distance function. High rates of mutation and lack of genome repair mechanisms in many viruses generate high levels of intraspecific diversity and result in quasi-species, particularly for many single-stranded RNA viruses. A recent survey of different phylogenic analysis methods has suggested that phylogenetic networks provide an alternative to phylogenetic trees and may be more suitable for data sets where evolution involves significant amounts of events such as hybridization, horizontal gene transfer, or recombination [28].

## Genomic Barcodes, Motif Fingerprinting and Genomic Signatures

The need for genomic-based microbial discrimination has been highlighted by multiple funding agencies for several years. However, widely acceptable standards concerning the naming conventions, length and coverage of genomic signatures to be used in biodefense applications, and the benchmarking of different approaches to generate them, have not yet emerged. In the literature, these discriminatory segments have been named pathogen signatures [24,29–31] and also DNA or genomic barcodes [32–34], or DNA fingerprints [35–38]. Despite the significant amount of literature, the use of this information for pathogen classification is limited because not all portions of a viral or bacterial genome are suitable for this type of phylogenetic analysis and therefore for classification. For example, DNA segments from ribosomal DNA are used as barcodes, however, this information does not correlate with phenotypical characteristics such as virulence or host range. Furthermore, researchers that use both neighbor-joining and maximum parsimony phylogenetic analyses have argued that DNA signatures and fingerprints do not contain sufficient information to build reliable phylogenic associations [39].

One approach, that in recent years has attracted the attention of taxonomists, is genomic barcoding. This method is based on a standardized genome segment used as the discriminatory parameter for detecting the presence of an organism and/or to distinguish it from all other species [24,40,41]. However, there are several discrepancies in the terminology used to define DNA or genomic barcodes. First, since these segments are not analogous to “barcodes”, they do not represent a priori manmade barcodes that can be used for identification. Several research groups have proposed that discriminatory segments should be named DNA fingerprints. However, in order to be considered fingerprints, DNA sequences must be identified as unique to a given taxonomic group. This requires an extensive sample collection effort, which is, at this moment, both not available and impractical for microbes of biodefense relevance. Furthermore, there is not a clear standard on the number of polymorphisms that should be used to establish the cutoff on species or strain separation and classify different fingerprints.

While between-species variations in DNA barcodes and fingerprints have been amply pointed out in the literature, systematic tests to determine the power of these signatures have been rare. Furthermore, some of these identification systems have been developed by considering a static version of a particular microbial genomic database. As a result, the rate of genomic erosion (specificity loss) or variant expansion due to the growth of the genomic databases is unknown. Consequently, despite their potential, there is disagreement about the usefulness of this approach for pathogen identification and characterization. This issue is more relevant in microbes with genome regions evolving at dissimilar rates and because the selected segments might not correlate mutations with epidemiological dynamics, disease severity, or geographical distribution. While pathogen genomic barcodes constructed using virulence factors have been proposed [42], in fact virulence is determined by a series of complex factors that also include the host. Considering the state of this technology, we propose a way to clarify the related terminology.

### Exogenous genomic barcodes and watermarks

In 2004, the JASON Defense Advisory Group proposed a DNA barcoding approach for tracking microorganisms by using exogenous DNA tags designed to be robust against mutations and inserted into the genome for detection and classification purposes. The overall barcode sequences were conceived to consist of three parts: a single central region containing a unique DNA barcode sequence with a previously known sequence pattern, flanked on either side by two shorter regions, designed to serve as universal primer sites for the PCR amplification of the central region. These types of artificial barcodes would serve as silent genetic markers consisting of a sequence of 20–36 base-pairs that could, in principle, generate from 1012 to 1021 unique serial numbers or barcodes. To prevent self-priming and false amplification during the PCR reaction, a barcode should not form a natural DNA hairpin structure. Furthermore, to remain biocompatible, the G:C content of the barcode should be roughly similar to that found in living organisms. The group also introduced the concept of genomic watermarks as DNA segments carrying information in a more distributed (holistic) manner and that are designed to be difficult-to-impossible to locate and read without special knowledge.

### Endogenous motif fingerprints and genomic signatures

This schema begins with the assumption that each species has distinctive short protein-coding genome segments that can be used for classification purposes. A motif is derived from the local alignment of a group of protein segments conserved by a minimum of 70% of sequence similarity. The exhaustive search overcomes the lack of sensitivity and specificity of conventional sequence analysis tools. It is possible to quickly sort unknown specimens into genetically different categories. Generally, the motifs do not overlap, but are separated along a sequence, though they may be contiguous in 3D-space [43]. The definition of motif fingerprint is used only when: a) the segment is specific to a given specific taxonomic group, and b) the discriminatory power of these motifs is defined by exhaustive database scanning. DNA fragments that code for a motif fingerprint are defined as genomic signatures. Motif fingerprints and genomic signatures can be used individually, or in the context of specific proteins and/or whole genomes, to yield insightful results about a specific pathogen’s origin, evolution and/or adaptation.

In contrast to other genome analysis approaches, endogenous motif fingerprinting and genomic signatures can uniquely classify members of a given taxonomical viral or bacterial group and provide the basis for understanding the relatedness of microbial strains at different levels of resolution. Thanks to this approach, it is possible to build a theoretical framework that can facilitate the development of new questions and experiments and expand the recognition of unknown variants using different detection and characterization technologies. For example, motif fingerprints and genomic signatures can be correlated with the emergence and geographical spread of virulent or drug-resistant pathogen variants, reservoirs and transmission modes. Also, motif fingerprints and genomic signatures can be considered as “molecular-operational taxonomic units” (M-OTU), where each M-OTU encompasses sufficient variability to allow both inter- and intra-species discrimination.

By scanning all the proteome of an organism, one can identify exogenous motif fingerprints and genomic signatures which are not limited to specific sequences of genes known to be involved in virulence and toxin production, but rather are focusing on the specificity to the pathogen species. This avoids the pitfalls of preconceived biological assumptions about virulence. By limiting the database to a set of genomic signatures specific to select agent sequences, the screening system minimizes false positives. Furthermore, this approach offers the possibility to develop robust combinatorial probability approaches where the presence of genomic signature combinations can provide detection and characterization strategies for unknown pathogen species identification. Linked with evidentiary information, this approach can track agents of concern either through direct metagenomic sequencing or by incorporating these moieties in detection platform and allows for rapid technological insertion.

Therefore, motif fingerprints and genomic signatures can be used in several strategies:

1. Since motif fingerprints and genomic signatures are specific to an agent of concern, they can be considered to be genomic units and their number can be used to quantify the risk of proliferation. The higher the number of genomic units used in a particular experiment or in the construction of a chimeric entity, the higher the biosecurity requirements to access, maintain and experiment with these variants.
2. Motif fingerprints and genomic signatures can be categorized at different levels of resolution, not only to discriminate species and strains, but combinations of these genomic elements could be used for a) identification (e.g., laboratories having similar samples or specific culture conditions); b) discrimination of lineage to recognize specific variants, including combinations of different species and ancestors; c) correlations with phenotypic characteristics; d) growth media characteristics.
3. A database of genomic signatures of all fully or partially sequenced genomes of select agent list pathogens can be constructed to identify the origin and/or closest relatives of segments of unknown DNA. However, implementations for searching genomic signatures must move away from tools seeking to determine the probability of finding such matches. Rather, sequence search algorithms must provide values independent to the search database space. This is due to the fact that the size of many sequence databases and bioinformatic applications might preclude proper detection and might yield a significant number of false negatives.
4. To be practical, DNA signatures must satisfy three criteria: a) contain significant species-level genetic variability and divergence; b) possess conserved flanking sites for developing universal PCR amplification primers; c) have sufficient length to be ubiquitously incorporated into different hybridization and amplification detection platforms.
5. Collective protection systems can add specific sets of genomic signatures to facilitate the integration of multiple data formats generated by different detection devices. With this information, it is possible to develop validation tests for false positive and false negative events and for determining sensitivity levels.
6. Motif fingerprints can be used to establish natural pathogen concentrations in the environment for environmental monitoring (e.g., areas for future troop deployments) and for future reference in forensics/attribution and restoration activities (e.g., cleaning up to the pre-existing environmental standard).
7. Recognizing genomic plasticity is dependent on association algorithms based on specific motif fingerprints that allow comparative analysis among databases and at the same time infer what proportion of the DNA sequence of ancestral and adapted strains exist.
8. Because their specificity and conservation to a given taxonomical group, therapeutic countermeasures minimizing off-target effects can be implemented with gene-to-drug and one-drug-many-bugs paradigms. These paradigms can be applied at the DNA level (e.g., siRNAs) or proteome level (e.g., therapeutic antibodies, small molecule targets and drugs).

### Other discrimination systems

Optical Mapping is a non- gel-based, PCR-based or sequencing-based approach for generating whole genome, high resolution restriction maps that can be used in strain typing, comparative genomics and whole genome sequence assembly. Nucleic acid-based discriminatory methods, including multiple-locus variable-number tandem repeat analysis (MLVA), multiple-locus sequence typing (MLST), 16S rRNA gene ribotyping, BOX-PCR fingerprinting, PCR-SSCP, DNA-DNA hybridization analyses and a variable amplicon typing (VAT) systems, have been used in pathogen characterization. However, thus far, truly reliable methods for confirmatory identification and characterization have not been standardized.

## Opportunities for Genomic-based Pathogen Classification

It could be argued that biosurveillance is one of the first lines of biodefense [44] and, at this level, genomic-based pathogen discovery and discrimination are fundamental components. However, the use of genomic information for pathogen characterization is complicated by the state of uncertainty that revolves around the fact that the inclusion of infectious microbes from a biodefense context considers DNA in a limited scope. It would be a profound mistake to see genomics as simply a scientific revolution and a component that cannot be part of the requirements of active pathogen monitoring. Therefore, there is an urgent need for device approaches that exploit genomic information, not only to provide regulatory clarity within the select agent list, but also to propose a list of pathogens of interest that the biodefense community must focus on in order to develop better biosurveillance approaches.

Since the detection content (primers, probes and antibodies) is updated as new sequences become available, surveillance systems must also upgrade their content design relatively frequently to accommodate this new genomic information. However, this task will be overwhelming and impractical, perhaps as soon as the end of 2012. By that time, one year of genomic data produced by unbiased next generation sequencing could double all the sequence information accumulated in the last decade, causing significant ‘confidence erosion’ in the ability of current tools to identify all variants of a signature. This genomic based perspective has significant challenges: How can specific genome regions be used to determine the agent’s origin? How can the possession of chimeras and genetically engineered pathogens indicate the offensive capacity of a country, or their intention to develop a biological weapons program? How do we track the transfer of pathogens from one laboratory to criminal organizations or individuals? What is the likelihood that a newly discovered human pathogen will become a pandemic?

Answering the above questions requires not only rethinking possible threat scenarios but developing new bioinformatic capabilities that address the specific and strategic operational needs of different biodefense stakeholders. This demands genomic-based risk stratification technologies that provide decision making information about the nature and risk of the threat and that rapidly proposes countermeasures that can be shared with a network of devices using DNA or amino acids as their recognition platform. While DNA sequencing technology is increasingly cost-effective for collecting large volumes of genomic and metagenomic data, the process of extracting meaningful conclusions to address biosurveillance needs remains muddled.

Unfortunately, current practices tend to favor uploading large volumes of genomic data into centralized analysis systems with limited capabilities for enforcing and promoting annotation, disambiguation and correction. As a result of this process, incorrectly annotated genomic records are propagated to other databases used to select and prioritize targets for countermeasures development. Furthermore, a centralized schema might result in a slow response when hundreds of samples from different locations are sequenced in a massive scale and need to be analyzed in parallel. As genome data accumulates, it is now evident that analysis will not be solved simply by scaling up the computational resources. As DNA sequence data continues to increase, the transfer and analysis of this data in near-real-time scales will become more pressing. Therefore, the development and application of tools that exploit decentralized enterprises for biodefense are necessary. For the short term, researchers must develop applications that exploit the minimum number of CPUs available in each bioinformatics center. As DNA technology performance increases, it is likely that biodefense researchers will be confronted with data streams of genomic information that require near-real time analysis systems.

While there is a diversity of bioinformatics techniques, the lack of benchmarking to properly assess the limitations of each tool and provide program managers with an assessment of the government investments is still lacking. This is limiting practitioners and policymakers in defining specific bioinformatics applications needed. Nonetheless, new theoretical and conceptual advances, that cope with the type of data in hand and the accuracy required for forensics attribution, are necessary.

Many bioinformatics tools are not scalable and it is not clear to what extent genomic and metagenomic information can be currently used to include new and unknown pathogens within a biodefense priority agent list. This problem revolves around three main questions: How can genomic information be used to classify and prioritize specific microbes and related species within a select agent list? What percentage of the genome, of a species related to a select agent list organism, can be used to form a chimerical organism without making it a select agent? Which is the best approach to exploit genomic information to generate standardized next generation detection platforms?

While there are several genomic data sets that can be used to test models and bioinformatic applications, it remains unknown what percentage of the genome of microbes within the select agent list are shared with other closely related microbe species and hosts genomes. This is of major significance for attribution and forensics, because the loss of specificity of a region cannot be used as evidence or for prosecution purposes. For example, chimeric viruses are useful for the study of viral replication and host factors that are involved in the transmission process. Currently, the National Center for Biotechnology Information (NCBI) stores genome information for more than 300 chimeric entries, including Dengue-2-Dengue-3, EBOV-Zaire-HIV [45], Alphavirus-Chikungunya [46], Argentine-Junin [47], H1N1-N5N1 [48], Dengue-Yellow fever (YFV) and tick-borne encephalitis virus (TBEV)-Japanese encephalitis virus (JEV). While one or both species forming the chimerical virus are listed within the select agent list, the chimera itself is not included and parameters for their inclusion are limited or nonexistent. Therefore, there is considerable uncertainty as to what constitutes a true ‘species’ in the microbial world. Nonetheless, researchers have rationalized the existence of constrained pathogen genome sequences that can be used for biothreat classification, attribution, forensics and prosecution [15,49]. Since the discovery of these segments remains elusive, there is the need to revise the quality and quantity of genomic information that is necessary to achieve the minimum discrimination resolution using that genomic information.

This uncertainty complication arose because of the frequent acquisition of foreign DNA via horizontal exchange by phage, transposons and other mobile genetic elements. Such chimerisms, generated by genome shuffling, prevail in several of the bacterial species on the select agent list, *Burkholderia* being particularly prolific in this regard [50,51].

## Implications for Synthetic Biology

Regulating the trade of specific sets of DNA sequences may reduce the risks associated with the misuse of synthetic life technology. On the one hand, gene synthesis companies produce a vast number of different gene sequences and the additional overheads of biosecurity screening could raise the costs of DNA synthesis [52]. However, if properly used, genomic signatures can play a significant role in the monitoring of DNA synthesis orders that use sequences of pathogens of concern [53–56]. As in synthetic biology, the use of pathogen genomic signatures can be considered a part of the design of a new biological entity. Therefore, an abstract view can be formulated where different parts are required for functioning. Such an approach could addresses concerns about the possibility of engineering *de novo* biological constructs with well-characterized input–output behaviours and interfaces. Furthermore, the development of consistent, standardized schemata for the representation of biological parts can allow the biodefense community to re-think new analysis strategies that will help the design of next generation detection technologies, combinatorial broad spectrum vaccines and therapeutics.

## Opportunities

In order to address the recommendations of previous HSPDs and NSB it is necessary to develop new genomic-based analysis algorithms that address the requirements and needs of different biodefense stakeholders. It now obvious that the decentralization of DNA sequencing has not been coupled by decentralized bioinformatics resources for biodefense. Decentralized genomic and metagenomic analysis architecture for “in-network knowledge discovery” should be developed in such way that can be used as test bed for new information analysis proving biodefense-oriented solutions. Such a system for biodefense is not only feasible, but will be more robust while fomenting crowdsourcing innovation and rapid response in to an unexpected event. This decentralized system can be defined as a collection of multiple information systems that synchronously cooperate via a communication network. This will require that biodefense stakeholders invest in developing strategies for computing workloads and ensure the availability of well-annotated databases. Therefore, DoD, DHS and NIH must continue to motivate performers in the value of annotating genomic records using the well-established GenBank format and must discourage the development of unique or “community” formats that in the end are difficult and expensive to maintain, upgrade and share.

In contrast to “cloud computing” where multiple units, none of which possess global knowledge, perform local data analysis a “biodefense in-network knowledge discovery system” should but cooperates in information discovery and sharing at the global and report summaries relevant at the local state. This will require new data analysis approaches that will support pathogen discovery, biosurveilliance and situational awareness for the warfighter. This knowledge approach can clearly be improved by additional enabling international nodes that exchange information with their constantly changing neighbours. A trend that could emerge is the use of extrapolation as a preliminary step to device genomic based pathogen risk assessment and to establish temporal states of information about a specific pathogen. If this state of belief reaches some threshold, more complex analysis algorithms or alerting system can be employed.

In the next three years, the “genomic revolution” will challenge analysis capabilities and will become data streams. Then data mining algorithms will require single pass, or a few passes to determine if biodefense relevant pathogens are present. The continuous, near real-time nature of the data streams requires data mining algorithms to continuous and on-the-fly. This situation will open the possibility to device firmware configurations for in situ pathogen discrimination. While these systems are unavailable because timing constraints for real-time data processing, hardware designs for operation under field conditions. This area could open new directions in data summary reporting.

## Eminent Danger

Anticipating and responding to biothreats requires the exploitation of genomic information in new ways to provide support to first responders, military commanders and other decision makers. While some argue the benefits of the genomic revolution, the complexity of this information and the limited capability of analysis tools highlight that one of greatest dangers is not communicating to the general population that they cannot be protected against every threat listed in the select agent list. Since it is unaffordable to protect against all biological threats, choices must be made. While risk-based allocation decision-making is still evolving, we must take a more systematic and reasoned approach to allocating resources for sequencing pathogens of biodefense relevance. Traditionally, concentration has focused on rapid response to the detection of new disease syndromes. However, metagenomic scale sequencing is showing us that we are surrounded by many pathogenic microbes [57–60].

## Conclusions

In the past few years the life sciences field has shifted into a landscape that has opened unexpected new paradoxes and dilemmas. On the one hand, genomic information can lead to better diagnostics, therapeutics and prophylactics. On the other, this data can be used with malevolent intent to design and engineer combinatorial pathogens that do not exist in nature. Therefore, to increase biosecurity, it is necessary to clarify the methodology for select agent list inclusion by using genomic information. In is now increasingly evident that the limiting stage in the study of pathogen diversity has shifted from the generation of data to the careful analysis and interpretation of genomic and metagenomic information. There is currently a dearth of high efficiency analytical tools which could consume the data in all of its raw forms to generate actionable and practical insights within a short time frame. However, for each search algorithm available for sequence analysis, a plethora of search parameters can be modified by the user. This situation generates sequence targets that lack standardization and complicate domestic and international pathogen surveillance efforts. Unless corrections are implemented to strengthen the standards for annotation of genomic data, sequence analysis algorithms will continue to have limited impact on biodefense and their capacity to alert us about unknown pathogens. While significant advances have been made, the biodefense community must develop additional new tools to generate genomic signatures and estimate risk and the inclusion of closely related microbes. Since biodefense laboratories are anticipated to offer more comprehensive sequence analysis, a genomic-based classification infrastructure, as well a forensic and intelligence system to collect, store, analyze, and interpret data generated by next generation sequencing, is required. This system must be scientifically robust and useful for tracking a sample from its isolation point, and it must be able to determine which institutions, and who within them, handled this material. In this regard, the selection of specific motif fingerprints and genomic signatures can provide not only discriminatory parameters for pathogen classification, but strengthen the regulatory framework of pathogens of interest for biodefense.

## Figures and Tables

**Figure 2:**
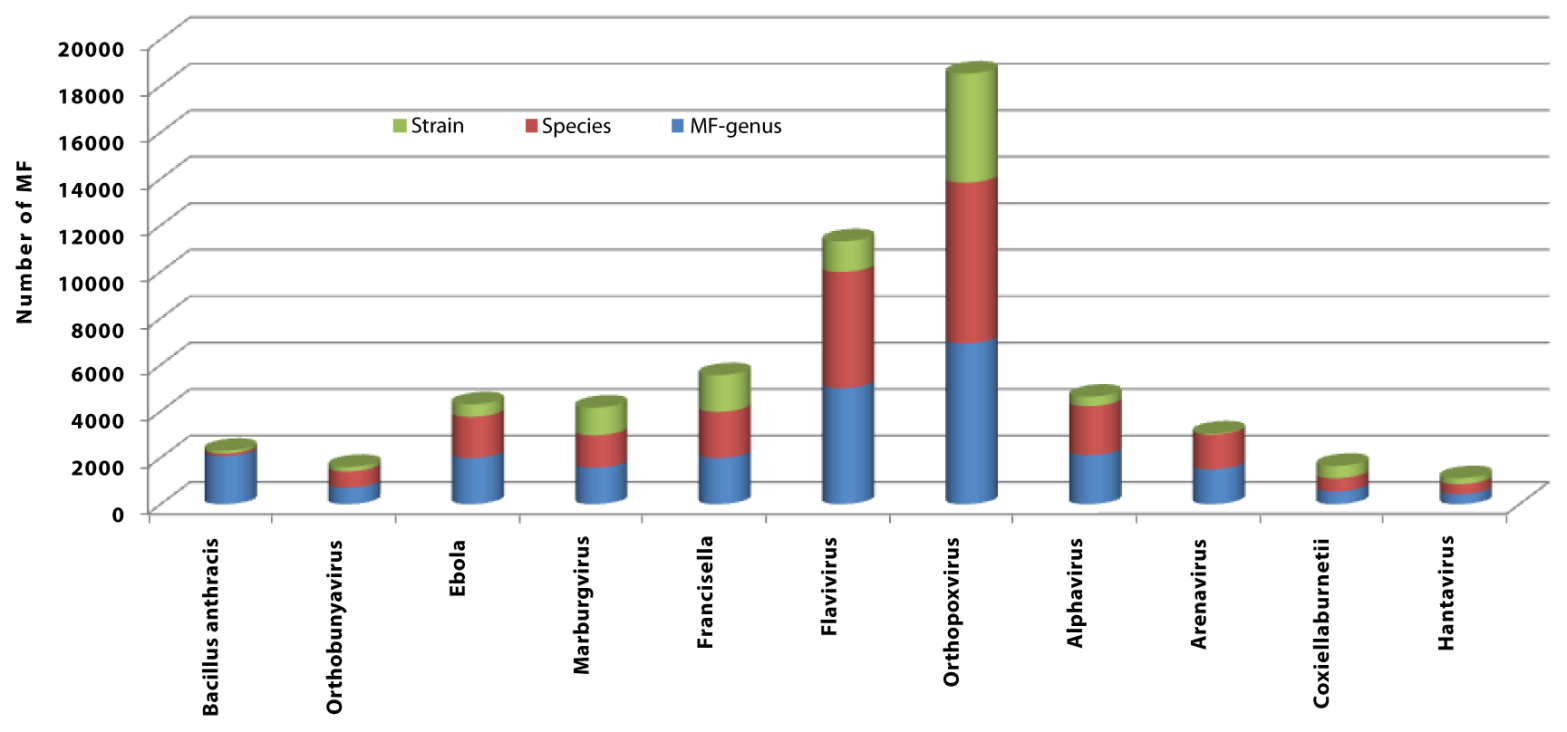
Number of Type I Motif Fingerprints from different pathogens of biodefense relevance.

**Figure 3:**
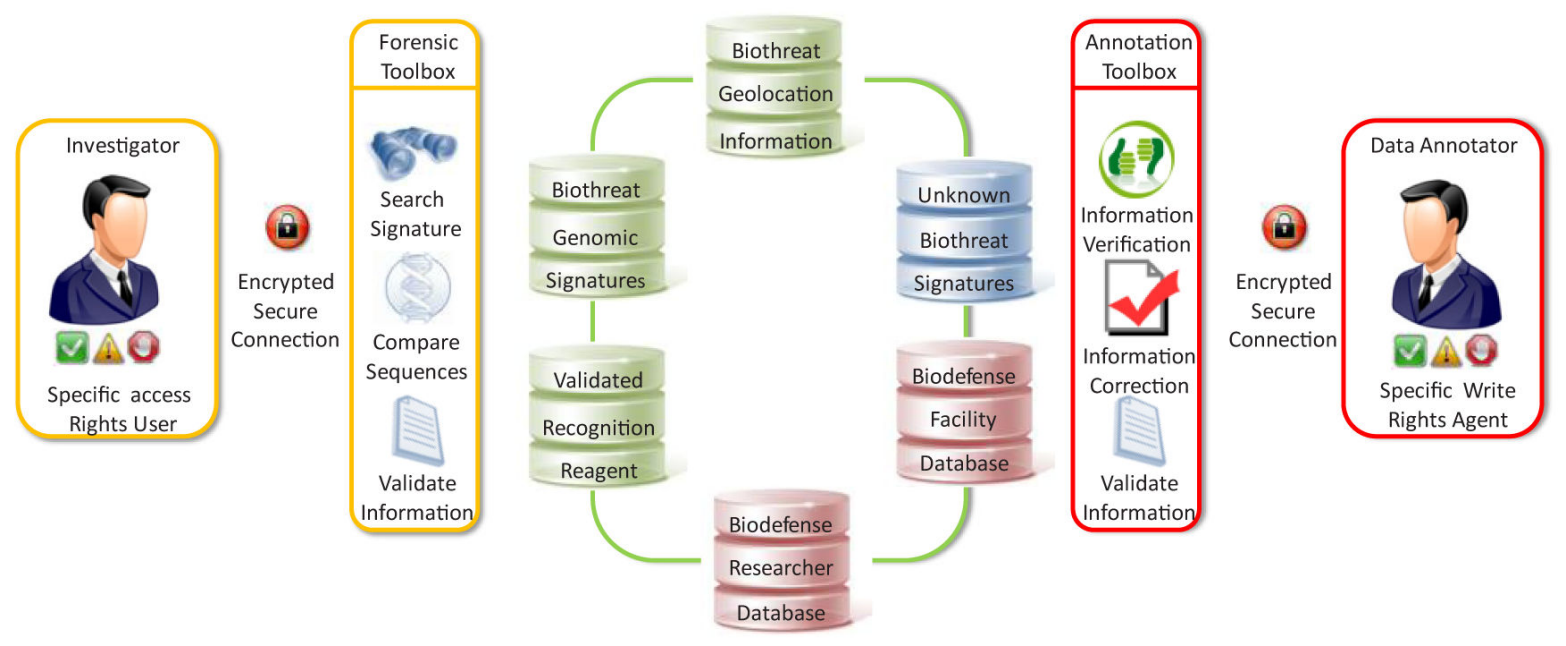
Schematic representation of a system to catalog select agent microbial collections Examples are shown of unclassified compliance systems dedicated to storing unclassified or “for official use only” (FOUO) biological threat information, as well as descriptions of research institutions, groups, or individuals accessing specific biological agents. There is an effort to create a global standard for reporting microbial collections which would include analytical and technological capabilities that can be reported under BTWC treaties.
